# A Cross‐Sectional Study of Patients Satisfaction With the Pharmacy Services at the 108 Military Central Hospital: Determinants and Implications

**DOI:** 10.1002/hsr2.70868

**Published:** 2025-05-26

**Authors:** Do Xuan Thang, Hoang Anh Tuan, Cuc Thi Thu Nguyen, Diep Tran Phuong, Le Thu Thuy, Trung Nguyen Duc

**Affiliations:** ^1^ Faculty of Pharmaceutical Management and Economics Hanoi University of Pharmacy Hanoi Vietnam; ^2^ Faculty of Pharmacy, The 108 Military Central Hospital Hanoi University of Pharmacy Hanoi Vietnam

**Keywords:** determinants, healthcare quality, patient satisfaction, pharmacy services, Vietnam

## Abstract

**Background and Aims:**

Patient satisfaction is a critical metric for assessing the quality of pharmaceutical services in healthcare systems. This study aims to build a questionnaire with various items to comprehensively evaluate patient satisfaction with pharmacy services at the 108 Military Central Hospital and to identify key influential factors in their perception.

**Methods:**

The cross‐sectional survey was conducted from August 2023 to October 2023. The face and content validity were assessed by experts. The questionnaire's internal consistency (Cronbach's alpha) and construct validity (exploratory factor analysis) were explored. Multivariate linear regression was used to seek determinants.

**Results:**

A total of 457 patients were included for synthesis. The final version of the questionnaire, which had 22 items grouped into four factors (attitude and professional capacity of pharmacy staff, accessibility and transparency, services performed, and infrastructure), showed high consistency and validity. General satisfaction was average (4.09 (SD = 0.628) out of a maximum score of five), with the factor having the lowest score of pharmacist consultants and drug prices despite being the leading national hospital. However, the two factors having the most substantial influence coefficient on satisfaction were the infrastructure (0.433), pharmacy attitude, and professional capacity (0.386).

**Conclusion:**

The findings based on the comprehensive questionnaire with 22 items grouped into four factors showed that there were available determinants that needed improvement. Enhancing the quality of the strongest impact aspects of patient satisfaction should be done comprehensively and synchronously. In the case of limited resources, prioritizing actions in phases of time could make them more actionable and beneficial.

## Summary

- Patients were not satisfied when the pharmacists had little time for consultation, did not explain how to store drugs, and the drug price might go beyond the payment ability of patients.
- Improving pharmacy infrastructure could be key to boosting overall satisfaction and addressing core patient concerns.
- Our findings suggest short‐, middle‐ and long‐term strategies to prioritize infrastructure upgrades, strengthen pharmacist consultation, or promote drug pricing assessment to improve patient satisfaction.

## 1 Background

Patient satisfaction is a core first step in building and evaluating healthcare systems in many countries. The term patient satisfaction in healthcare was addressed in several theories, one of which involves a complex mixture of the patient's perceived needs, expectations, and actual experiences with the healthcare system [1, 2]. This multidimensional concept includes both medical and nonmedical aspects of health care. Thereby, many patient satisfaction assessments in various aspects of medical care were conducted in many nations to offer providers valuable insights into healthcare services, including the effectiveness of their care and the extent of empathy they exhibit [3]. The weaknesses in medical examination and treatment were also explored, and strategies for optimizing the quality of healthcare services were proposed from the studies. During this healthcare process, the hospital pharmacy is the final step, which releases all dissatisfaction accumulated from prior progress. With its vital role within the multidisciplinary‐integrated system, hospital pharmacy has many direct influences on patient satisfaction and the hospital's overall reputation [4]. High‐quality services' needs were especially increased through social information development, changes in client expectations, and increased competitiveness and cost in the health sector [5, 6].

Thereby, hospital pharmacies should pay attention to delivering high‐quality services that address patients' needs effectively. The service quality could refer to issues, including communication, signposting, information provision, and staff interaction with patients [1]. In patient‐centered health care, the pharmacist is expected to have an appropriate caring attitude and ensure that people can access medicines or pharmaceutical advice easily to enhance patients' health and overall well‐being [7, 8].

Many studies have been done to assess patient satisfaction with hospital pharmacy services [3, 9, 10], but the application may not be synchronized because of the different situations in each country. In Vietnam, health insurance coverage was applied nationally, the public hospitals were classified into three tiers (central, provincial, and district levels), and patients have a copayment ranging from 5% to 20% with the health insurance department [11]. Under the high pressure of financial autonomy, public hospitals compete highly with private hospitals to attract patients for examination and treatment. As a result, public hospitals must constantly improve the quality of service to satisfy patients, trust the hospitals, adhere to medication regimens, and have better health outcomes. Some studies on pharmacy service satisfaction were carried out [11, 12, 13, 14, 15]. However, they were from an available well‐defined theoretical model (SERVQUAL instrument) focusing on service quality across five dimensions [14], not based on patients' views [13, 15], assessed among community pharmacies [11], or just one polyclinic [12].

The 108 Military Central Hospital in Vietnam is a national multi‐specialty hospital and serves as the top‐tier hospital of the Military Medical Hospital, with over 2000 prescriptions per day, placing significant pressure on the Pharmacy Department. Despite the vital role of patient satisfaction in shaping the quality of healthcare services, there needs to be more research focusing on patient satisfaction towards the hospital pharmacy, especially among these leading national hospitals. A study at a Tertiary Care Center in the Kingdom of Saudi Arabia showed that many patients were satisfied with the pharmacy [16]. Still, a study at a specialized hospital in Ethiopia illustrated that outpatients' satisfaction towards pharmacy services was very low [17]. This difference could be explained by the fact that patients' expectations relating to these hospital pharmacies might be diverse on many sides [7]. Therefore, this study addressed gaps in this emerging area by assessing patient satisfaction with the hospital pharmacy from the synthesis questionnaire in many aspects and seeking to determine the determinants of patient satisfaction. Based on the findings, the recommendations proposed in the study can be adapted to suit the healthcare context of other hospitals in Vietnam, or even other countries.

## 2 Methods

### 2.1 Development of the Outpatient Satisfaction Evaluation Questionnaire

A satisfaction questionnaire was built based on the literature search in Pubmed during 2023 and the synthesis of existing validated instruments from the studies assessing patient satisfaction with the hospital pharmacy. A total of 15 studies that met the inclusive criteria were synthesized.

Face and content validity was verified by an expert panel, consisting of two pharmacists in academia (DXT and NDT), and one hospital pharmacist (HAT). Each item was reviewed, and the relevance and appropriateness of each item were discussed until the questionnaire covered all the important domains of patient satisfaction in hospital pharmacy. After the discussion, 28 questions were used, three were deleted, four were rephrased, and two new questions were added. The final version with 28 questions was grouped preliminarily into five domains based on the previous studies [7, 14, 18, 19], its relevance to the overall experience and quality of the pharmacy service and practical situations of hospitals to ensure comprehensive coverage, including accessibility, transparency of information and procedures for purchasing drugs, infrastructure and appearance of the pharmacy, pharmacist attitude and competence, and services performed at the pharmacy). One question relating to general satisfaction was also included (Table 1).

**Table 1:** The contents of the questionnaire.

No.	Code	Contents
Accessibility		
1	A1	The pharmacy was located at a convenient location
2	A2	Directions to the pharmacy were clear
3	A3	The pharmacy image and design were impressive and friendly
Transparency of information and procedures for purchasing drugs		
4	T1	The instructions on how to buy the medicine were posted clearly, easily to read and understand
5	T2	The medicine bill was fully provided
6	T3	The fairness of the cost of medications in the pharmacy
7	T4	Receive medications within a reasonable time
Infrastructure and appearance of the pharmacy		
8	I1	The pharmacy space is adequate
9	I2	The waiting area is clean and comfortable
10	I3	Enough seats were furnished in the waiting area
11	I4	The speaker system is clear and easy to hear
12	I5	The process of drug supplying was organized scientifically and appropriately
13	I6	Having a private area for consultation
Pharmacist attitude and competence		
14	P1	Having a good appearance and costume
15	P2	The pharmacy staff treat with respect
16	P3	The pharmacist showed readiness to listen to the query
17	P4	The pharmacist showed readiness to answer questions
18	P5	The pharmacist had the ability to answer questions and provide professional advice
19	P6	The clarity of the professional instructions from pharmacists about how to take your medication
20	P7	Careful and double‐checking was applied when delivering medicine
Services performed at the pharmacy		
21	S1	The right medicines were provided
22	S2	Medication quantity was supplied
23	S3	Medication appearance and quality were good
24	S4	The drug benefits were introduced
25	S5	Full instruction on medicines was provided
26	S6	The pharmacist explained how to store medication
27	S7	The pharmacist spends a reasonable amount of time responding to the needs and providing advice
28	S8	Total prescription value in line with ability to pay
Others		
29	GS	Satisfaction with service quality at the hospital pharmacy

All responses were measured on a 5‐point Likert scale, ranging from strong satisfaction to strong dissatisfaction. The general satisfaction with the hospital pharmacy were also assessed on a 5‐point scale. Moreover, the characteristics of patients (age, gender, ethnicity, occupation, educational status, marital status) and the characteristics of the disease (hospital visit frequency, diagnosis disease, the average time to buy medicine, and the re‐examination schedule) were collected.

### 2.2 Validation of the Questionnaire

#### 2.2.1 Study Design and Subjects

A cross‐sectional survey was conducted among patients or relatives who were adults above 18 years old and who purchased the drugs at the 108 Military Central Hospital pharmacy from August 2023 to October 2023. The exclusive criteria did not agree to participate in or did not complete the full survey.

#### 2.2.2 Sample Size and Sampling Procedure

The sample size for this study was determined by assuming a 50% satisfaction level with pharmacy services, as no prior research had been conducted on this topic at the Military Hospital. The sample size calculation used a marginal error of 5%, a $z_{1‐\alpha /2}$ value of 1.96, and a 95% confidence interval. From this information and using the single proportion formula, the sample size was calculated to be at least 384. This sample size also met the sample size based on 1:10 participants per item ratio to perform factor analysis with the minimum number of participants required.

The data was collected using a convenience sampling technique. The researchers explained the study's objectives to eligible participants and requested them to write informed consent before entry into the face‐to‐face interview for those who agreed to participate. All answers were coded, and patient information was deidentified.

The Institutional Research Ethics Review Board approved this study under No. 2311/PCT‐HDDD dated 05/08/2023, and the survey was carried out from August 2023 to October 2023 at the hospital pharmacy of the 108 Military Central Hospital.

#### 2.2.3 Statistical Analysis

The questionnaire's internal reliability was evaluated using Cronbach's alpha and corrected item‐total correlation. Cronbach's alpha value is greater than 0.6 and corrected item‐total correlations above 0.3 indicated acceptable internal consistency for each domain. Exploratory factor analysis (EFA) was used to assess the construct validity of the questionnaire and confirm the relevance of selected variables [12, 14, 20, 21, 22]. The Principal Component Analysis method was chosen for factor extraction, following these criteria: a Kaiser–Meyer–Olkin (KMO) value of at least 0.5, a Bartlett's test *p* value less than 0.05, an Eigenvalue over 1.0, a total variance explained greater than 50%, and the factor loadings at least 0.5 or higher, with Varimax rotation and Kaiser Normalization used [14, 20, 21, 22]. Multicollinearity effects to ensure no significant multicollinearity among the independent variables were calculated using variance inflation factors (VIF), which were found to be below two, indicating no multicollinearity issues. Multiple regression analysis was applied to determine the influential factors on outpatient satisfaction, with a *p* value of less than 0.05 considered statistically significant. All statistical analyses were done via R version 4.4.0.

## 3 Results

No further changes were recorded during face validity; the questionnaire was used for a total of 537 customers, of which 475 agreed to participate in the study (response rate of 85%).

### 3.1 Characteristics of Respondents

Characteristics of respondents are presented in Table 2. More than half of the participants were female (59.4%) and were married (83.2%). The average age was 43.11 (SD = 13.173). A total of 65.4% of the people surveyed were patients, and the rest were patients' relatives. Most were freelance businesses (27.1%) and had university or postgraduate degrees (35.4%).

**Table 2:** Characteristics of the outpatients.

Items	Categories	Number (%)
Age (mean (SD))		43.11 (13.173)
Gender	Male	172 (37.6)
	Female	273 (59.4)
Relationships with patients	Patient	299 (65.4)
	Patients' relatives	148 (32.4)
Occupation	Government official	100 (21.9)
	Worker	66 (14.4)
	Farmer	58 (12.7)
	Freelance business	124 (27.1)
	Retired	52 (11.4)
	Unemployment	4 (0.9)
	Other	53 (11.6)
Level of education	Secondary school or lower	36 (7.9)
	High school	165 (36.1)
	College	63 (35.4)
	University/post‐graduate	162 (35.4)
Marital status	Married	380 (83.2)
	Single	67 (14.7)
	Divorced/widower	6 (1.3)

### 3.2 The Mean Score of Items

**Figure 1:**
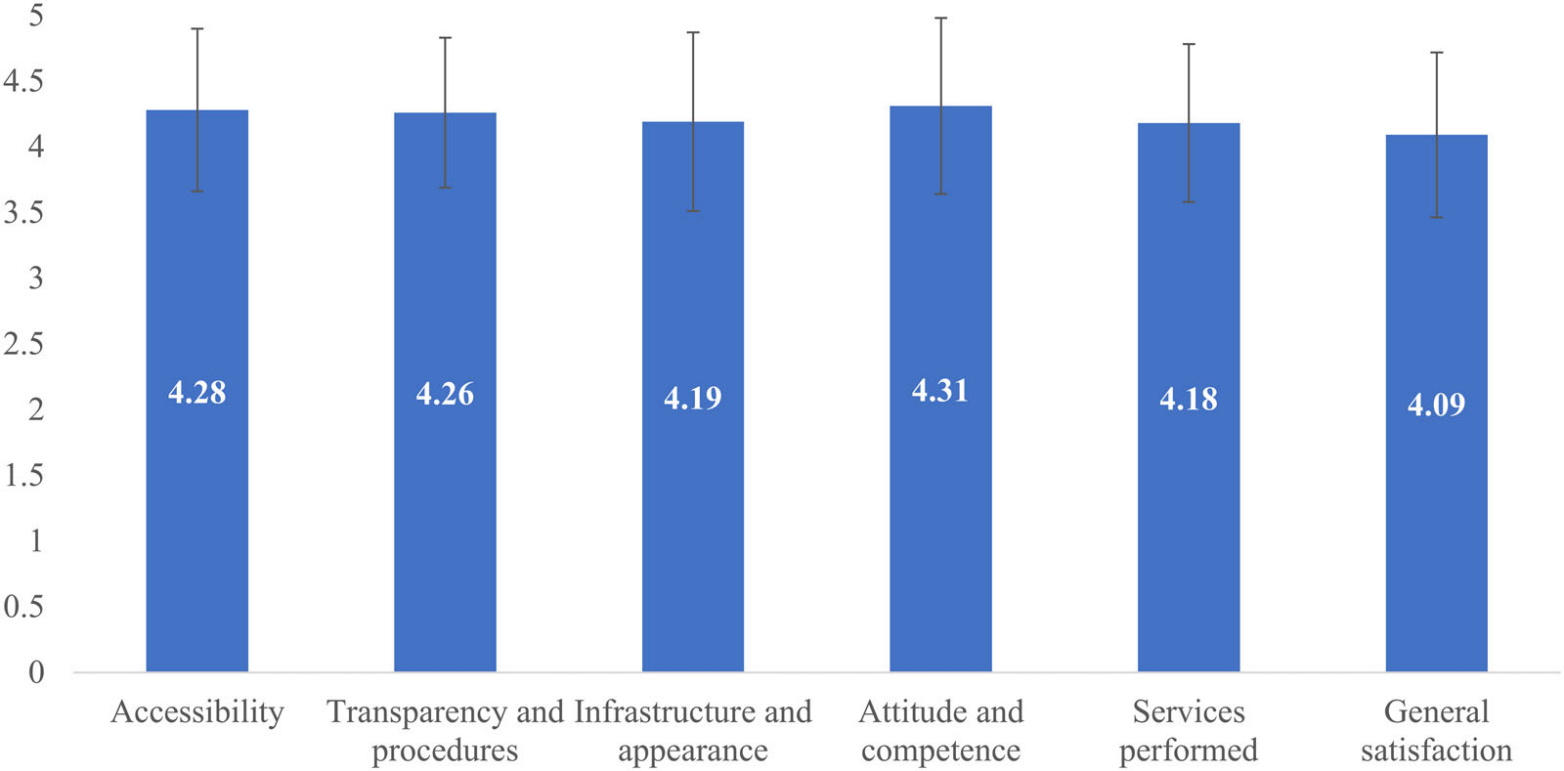
Score of patient satisfaction of domains.

The overall mean score of satisfaction with pharmacy services was 4.09 (SD = 0.628) out of a maximum score of 5. The mean score of each domain was also over 4.00 (Figure 1), with the highest domain being the pharmacist's attitude and competence (4.31, SD = 0.669) and the lowest one being service performance (4.18, SD = 0.602).

**Figure 2:**
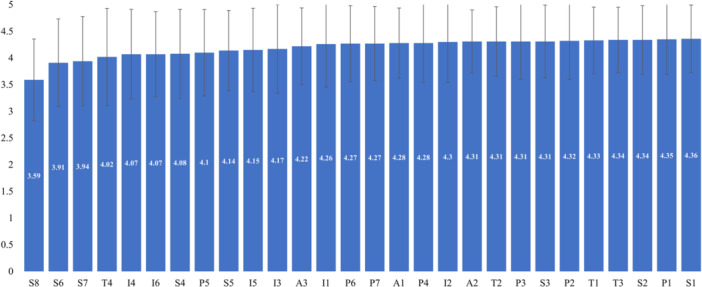
Score of patient satisfaction with items.

Among items of the domain with the lowest score (Figure 2), the item of total prescription value in line with the ability to pay got the lowest score (item code S8), followed by the pharmacist explaining how to store the medication (item code S6). These items also got the lowest scores among sub‐domains. The items with the highest scores were S1 (right medicines were provided), P1 (pharmacists had a good appearance and costume), and S2 (medication quantity was supplied), respectively.

### 3.3 Reliability and Validity of the Scale

Regarding internal reliability, the results in Table 3 showed that all scales of the five‐factor groups had a Cronbach's Alpha coefficient of over 0.6, and the corrected item‐total correlation of each variable was greater than 0.3. Thus, the scale met the reliability requirements.

**Table 3:** The questionnaire's validity and reliability analyses, considering the mean score for patient satisfaction for each domain and its items.

No	Domain	Code	Cronbach's alpha	Corrected item—Total correlation	Cronbach's alpha if item is deleted
1	Accessibility	A1	0.910	0.852	0.843
2		A2		0.872	0.872
3		A3		0.898	0.898
4	Transparency and procedures	T1	0.845	0.749	0.781
5		T2		0.800	0.757
6		T3		0.818	0.756
7		T4		0.488	0.927
8	Infrastructure and appearance	I1	0.884	0.786	0.849
9		I2		0.845	0.841
10		I3		0.704	0.863
11		I4		0.633	0.877
12		I5		0.649	0.872
13		I6		0.588	0.881
14	Attitude and competence	P1	0.956	0.743	0.958
15		P2		0.875	0.947
16		P3		0.916	0.944
17		P4		0.910	0.945
18		P5		0.754	0.959
19		P6		0.877	0.947
20		P7		0.888	0.947
21	Services performed	S1	0.885	0.702	0.868
22		S2		0.724	0.866
23		S3		0.724	0.865
24		S4		0.727	0.863
25		S5		0.740	0.862
26		S6		0.639	0.873
27		S7		0.671	0.870
28		S8		0.381	0.898

The final 28 observational variables were evaluated as EFA with the further elimination of six observed variables (Table 4). The remaining 22 variables were validated into the four‐factor model, with the results as follows: KMO of 0.930, Barlett's test of sphericity less than 0.05, Eigenvalue of all factor groups were greater than 1, Factor loadings higher than 0.5, and the total variance extracted of 74.55%. Thereby, a four‐factor model comprising of attitude and professional capacity of pharmacy staff, accessibility and transparency, services performed, and infrastructure were valid and there was no need for readjustment.

**Table 4:** Exploratory factor analysis.

Items	New factors			
	Attitude and professional capacity of pharmacy staff	Accessibility and transparency	Services performed	Infrastructure
P4	0.861			
P3	0.848			
P6	0.820			
P7	0.816			
P2	0.802			
P5	0.741			
P1	0.601			
A1		0.855		
A3		0.811		
A2		0.810		
T1		0.702		
T2		0.670		
T3		0.658		
S6			0.826	
S7			0.816	
S5			0.754	
S4			0.611	
S8			0.572	
I3				0.828
I2				0.786
I4				0.785
I1				0.663
Eigenvalues	11.452	2.101	1.653	1.193
KMO	0.930			
*p* value in Bartlett test	< 0.001			
Total variance explained	74.55%			

### 3.4 Multiple‐Regression Analysis

The results of VIF calculation and multiple‐regression model analysis were illustrated in Table 5.

**Table 5:** VIF indicator and multiple‐regression analysis.

Factor	*B*	*β*	Sig.	VIF
Intercept	4.090			
Attitude and professional capacity of pharmacy staff (X_1_)	0.386	0.333	0.000	1.000
Accessibility and Transparency (X_2_)	0.332	0.287	0.000	1.000
Services performed (X_3_)	0.106	0.092	0.017	1.000
Infrastructure (X_4_)	0.433	0.374	0.000	1.000
*R* ^2^	0.342			
Adjusted *R* ^2^	0.336			
Durbin–Watson	0.566			
*p* value ANOVA	< 0.001			

All factors had a statistically significant influence on customer satisfaction (*p* values < 0.05). The adjusted *R*‐squared was 0.336, indicating the explanation of the model of 33.6% of the variation in satisfaction with no autocorrelation (Durbin–Watson value less than 2). The regression model was statistically significant (ANOVA *p* value < 0.001), and the multicollinearity was not detected (all VIF values less than 2). Additionally, all independent variables demonstrated a consistent positive relationship with the general patient satisfaction, with positive coefficients. Infrastructure had the strongest impact (0.433), followed by pharmacy staff's attitude and professional capacity (0.386). On the other hand, positive β coefficients for each variable, especially the highest values of infrastructure and pharmacy staff's attitude and professional capacity, showed that improving these factors had a statistically substantial effect on increasing customer satisfaction. The regression equation was:

$$\text{GS}\;=4.090+0.386{*X}_{1}+0.332{*X}_{2}+0.106{*X}_{3}+0.433{*X}_{4}$$

## 4 Discussion

Our results demonstrated that four components affected patient satisfaction with pharmacy service. The highest mean score was in the accessibility domain, with a mean score of 4.28, reflecting high satisfaction among respondents with the convenience, clear signage, and overall image of the pharmacy department. In contrast, the lowest mean score was service performance. Among the items of service performance, pharmacists' consultation and the drug price were the lowest; patients were not satisfied when the pharmacists had little time for consultation, did not explain how to store drugs, and the drug price might go beyond the payment ability of patients. These results were similar to other studies in Spain [23], Nigeria [24], and Ethiopia [25]. In the provision of pharmaceutical services, the consultant services of pharmacists play an important role in reducing medication errors and enhancing treatment effectiveness [26], even contributing to the cost‐effectiveness of all healthcare systems across various settings [27]. Though the 108 Military Central Hospital in Vietnam is a leading national hospital, the pharmacists still showed shortcomings in patient counselling. This result showed the lack of rigour in implementing training, assessment, and improving knowledge for pharmacists at this hospital level. The lack of time for consultancy is understandable with the huge number of prescriptions per day, but it also simultaneously shows great pressure and high workload on the pharmacy department, which easily causes a large number of medication errors [28]. Thereby, it is necessary to have an improvement strategy to create positive and meaningful patient–pharmacist communication [10, 29], and to increase the number of pharmacists at the dispensing window, which can enhance patient throughput while decreasing the workload on individual pharmacists [28]. In addition, training and knowledge assessment should be carried out regularly to ensure that pharmacists have the updated knowledge and necessary skills to communicate and counsel patients, especially in large‐scale hospitals.

Regarding drug prices, Vietnam's healthcare model requires the promotion of using domestically produced drugs or generic drugs with much lower prices than brand‐name drugs; however, patients were not most satisfied with paying for drugs. It could be explained by the fact that in Vietnam, patients have a copayment ranging from 5% to 20% with the health insurance department, and different levels of diseases require various treatments and result in dissimilar medical expenditures. Besides, not all drugs are reimbursed by health insurance, and such large medical expenditures may seriously affect mental health and decrease the quality of healthcare [30]. However, the rigour and reasonability of prescriptions also need to be assessed when many unnecessary drugs, such as supplement drugs, might be prescribed, or brand‐name drugs with high drug costs are routinely prescribed. Consequently, further studies should be conducted.

Moreover, the preliminary two factors of accessibility and transparency and the procedures were grouped into one factor after running EFA; it showed that there was overlapping content or similar perceiving of the patients. This highlighted the interaction between the theoretical framework based on the synthesized questionnaire and the reality of patient perceptions. This finding was similar to the previous study [31]; it provided a foundation to offer insights on refining and restructuring survey instruments, exploring higher‐order constructs, and improving their validity and reliability for use in pharmacy practice. These insights can lead to more precise measurements and real recommendations for improving patient satisfaction.

General satisfaction reached a mean score of 4.09, further supporting the overall positive evaluation of pharmacy services. Since the other domains had a lower score, it suggests a need to align between specific elements of a service and customer expectations to strengthen overall satisfaction. This result was similar to previous studies [3, 32] and highlighted the significant efforts to be taken to enhance the levels of patient satisfaction.

Lastly, our multiple regression analysis revealed that all four factors had a statistically significant association with patient satisfaction. Though patients expressed the lowest satisfaction with the services performed, the factor with the highest level of influence coefficient was the facilities. This result implies that counselling and pricing are important, but facility maintenance is more significant in determining patient satisfaction. Well‐maintained and proper facilities would raise the patient experience by ensuring comfort, accessibility, and efficient delivery service. Consequently, improving pharmacy infrastructure could be key to boosting overall satisfaction and addressing core patient concerns. This finding was also aligned with the WHO's suggestion that infrastructure and human resources affected the quality of care, and they were the key points for high‐quality services [19]. Many patients may seek healthcare facilities through referrals from others or even from their initial impressions of the hospital, so initial impressions of the physical environment may shape subsequent patient satisfaction [33]. Moreover, some studies [34, 35] showed that healthcare staff had enhanced communication with outpatients in smaller hospitals because of more time and lower pressure. However, communication decreased among normal‐large hospitals and became worse among medium‐large hospitals because outpatients' perceptions and expectations of the quality of professional services in the hospital increased significantly, while the healthcare staff had higher workloads and pressure. However, if the scale of the hospital is expanded to an extra‐large level, outpatients would pay more attention to facilities and the environment. These new points were consistent with our results and suggested a suitable strategy for hospital administrators based on the scale of the hospital.

Though many problems should be paid attention to in comprehensive and synchronous improvement, the limited resources require prioritizing the different strategies. The first short‐term plan should be to open training courses to enhance the communication between pharmacists and patients and improve pharmacists' knowledge and consultation skills. Then, the medium‐term strategy may boost the infrastructure upgrades, broaden the dispensing window, and improve the procedure to decrease waiting time. Finally, the long‐term direction can be the market competition on drug prices and improved indication awareness, with pharmacoeconomic studies providing relevant evidence to persuade drug unit price reductions and eliminate unnecessary prescribing. This prioritization of actions across periods could make them more actionable and beneficial.

### 4.1 Strengths and Limitations of the Study

With the synthesis from many relevant studies to seek the items reflecting patient satisfaction, our study had a comprehensive number of items, allowing a full assessment of factors affecting patient satisfaction, especially in hospital pharmacy. The findings found the determinants affecting patient satisfaction with pharmacy services, and those were the basis for further interventions to improve and enhance the pharmacy services, and even all health and medical services of the hospital. Furthermore, the strong internal consistency across items and construct validity confirmed the promotion of the measurement model used, supporting its applicability in evaluating pharmacy service quality. There were some limitations to this study. First, data collected during a period of 3 months in 2023 may probably not fully reflect long‐term trends in the classification of disease across the year and may lead to a lack of the generalizability of results. Second, there was not much classification when assessing satisfaction, so there was not much distinction to detect satisfaction differences among patient subgroups, such as patients with or without difficult physics or worse diseases. The absence of subgroups may cause a potential bias, as aggregated results might mask important variations in patient experiences. As a result, a study with a longer period incorporating more subgroups could help mitigate this issue and improve the robustness of the findings.

## 5 Conclusion

The synthesis questionnaire, which was built with the final 22 items grouped into four factors, showed high validity in evaluating patient satisfaction with pharmacy services. General satisfaction was average, with the factor having the lowest score of pharmacist consultants and drug prices, despite being the leading national hospital. However, the factor that had the strongest influential coefficient on satisfaction was infrastructure. Depending on the country's context and the hospital's condition, these findings suggest short‐term and long‐term strategies. Policymakers and hospital administrators should prioritize facility improvements, prefer pharmacy consultation, or promote drug pricing assessment. By implementing these changes, patient satisfaction can be improved significantly, ensuring effective and patient‐centered healthcare delivery.

## Author Contributions

**Do Xuan Thang:** methodology, writing – original draft, writing – review and editing, conceptualization, and supervision. **Hoang Anh Tuan:** formal analysis, data curation, resources, and investigation. **Cuc Thi Thu Nguyen:** methodology, writing – original draft, writing – review and editing, validation. **Diep Tran Phuong:** data curation, formal analysis, and investigation. **Le Thu Thuy:** data curation, resources, and investigation. **Trung Nguyen Duc:** conceptualization, project administration, and supervision.

## Ethics Statement

This study was conducted in accordance with ethical standards and approved by The Institutional Research Ethics Review Board under No 2311/PCT‐HDDD dated 05/08/2023. The researchers explained the study's objectives to eligible participants and requested them to write informed consent before entry into the face‐to‐face interview for those who agreed to participate. All answers were coded, and patient information was deidentified.

## Conflicts of Interest

The authors declare no conflicts of interest.

## Transparency Statement

The lead author, Do Xuan Thang, affirms that this manuscript is an honest, accurate, and transparent account of the study being reported; that no important aspects of the study have been omitted; and that any discrepancies from the study as planned (and, if relevant, registered) have been explained.

## Data Availability

The data that support the findings of this study are available from the corresponding author upon reasonable request.
